# Cardiac autonomic control and complexity during sleep are preserved after chronic sleep restriction in healthy subjects

**DOI:** 10.14814/phy2.13197

**Published:** 2017-04-13

**Authors:** Eleonora Tobaldini, Naima Covassin, Andrew Calvin, Prachi Singh, Jan Bukartyk, Shiang Wang, Nicola Montano, Virend K. Somers

**Affiliations:** ^1^ Department of Internal MedicineFondazione IRCSS Ca' GrandaOspedale Maggiore PoliclinicoMilanItaly; ^2^ Department of Clinical Sciences and Community of HealthUniversity of MilanMilanItaly; ^3^ Department of Cardiovascular MedicineMayo ClinicRochesterMinnesotaUSA

**Keywords:** Autonomic nervous system, complexity, heart rate variability, sleep deprivation, symbolic analysis, sympathetic

## Abstract

Acute sleep deprivation (SD) alters cardiovascular autonomic control (CAC) and is associated with an increased risk of cardiovascular disorders. However, the effects of partial SD on CAC are unclear. Thus, we aimed to investigate the effects of partial SD on CAC during sleep. We randomized seventeen healthy subjects to a restriction group (RES,* n* = 8, subjects slept two‐thirds of normal sleep time based on individual habitual sleep duration for 8 days and 8 nights) or a Control group (CON,* n* = 9, subjects were allowed to sleep their usual sleep time). Attended polysomnographic (PSG) studies were performed every night; a subset of them was selected for the analysis at baseline (day 3‐D3), the first night after sleep restriction (day 5‐D5), at the end of sleep restriction period (day 11‐D11), and at the end of recovery phase (day 14‐D14). We extracted electrocardiogram (ECG) and respiration from the PSG and divided into wakefulness (W), nonrapid eye movements (REM) sleep (N2 and N3) and REM sleep. CAC was evaluated by means of linear spectral analysis, nonlinear symbolic analysis and complexity indexes. In both RES and CON groups, sympathetic modulation decreased and parasympathetic modulation increased during N2 and N3 compared to W and REM at D3, D5, D11, D14. Complexity analysis revealed a reduction in complexity during REM compared to NREM sleep in both DEP and CON. After 8 days of moderate SD, cardiac autonomic dynamics, characterized by decreased sympathetic, and increased parasympathetic modulation, and higher cardiac complexity during NREM sleep, compared to W and REM, are preserved.

## Introduction

Acute sleep loss is associated with an increased risk of developing cardiovascular diseases, cerebrovascular disorders, metabolic disorders (Eguchi et al. 2008; Cappuccio et al. 2010), and infectious diseases (Patel et al. 2012).

From a pathophysiological point of view, sleep loss is associated with activation of several molecular and integrative regulatory mechanisms, such as disruption of the autonomic nervous system (ANS) (Tasali et al. 2008; Tobaldini et al. 2013b, 2014), an impaired immune and inflammatory response (Imeri and Opp 2009).

The ANS plays a key role in linking sleep deprivation with detrimental clinical consequences of sleep loss. In fact, it has been shown that after 24 h of complete sleep loss, heart rate (HR) and arterial blood pressure (ABP) were higher compared to baseline in healthy subjects (Zhong et al. 2005; Sauvet et al. 2014; Sunbul et al. 2014). As to the ANS, sleep deprivation reduced total heart rate variability (HRV) (Zhong et al. 2005), which is considered a marker of the ability of ANS to respond to stress stimuli. An important change in the sympatho‐vagal balance, in terms of increased sympathetic component and reduction in vagal modulation (Zhong et al. 2005; Sauvet et al. 2014) has been described in healthy subjects. In addition, acute sleep loss blunted the haemodynamic and autonomic responses to a tilt table test, with a lower increase in HR, ABP, and sympathetic modulation in response to orthostatic stress in resident physicians after 24 h of acute sleep deprivation (Tobaldini et al. 2013b).

On the contrary, few data are available on the effects of chronic sleep restriction in healthy subjects, with conflicting results, partially attributable to the variety of experimental protocols applied. Two studies on the effects of 5 days of chronic sleep curtailment showed a reduction in total HRV and a marked sympathetic predominance after the deprivation period (Muenter et al. 2000; Dettoni et al. 2012). However, Muenter et al. (2000) found no differences in ANS control after chronic sleep loss. More recently, a paper reported that 1 week of chronic sleep restriction (4 h of sleep per night) induced an alteration of endothelial‐dependent vasodilation, independent of autonomic profile, but associated with activation of inflammatory and metabolic pathways (Sauvet et al. 2015).

Sleep is a physiological process characterized by profound changes of autonomic cardiovascular control during different sleep stages. As sleep becomes deeper, during NREM sleep, sympatho‐vagal balance shifts toward a vagal respiratory predominance, while during REM sleep sympathetic modulation becomes predominant, with repetitive bursts of sympathetic overactivity that may exceed levels manifested during wakefulness (Somers et al. 1993; Vanoli et al. 1995; Trinder et al. 2001; Brandenberger et al. 2003).

Over the past decades, growing interest has focused on the assessment of autonomic cardiac control during sleep using different tools, such as linear spectral analysis and, more recently, symbolic analysis and entropy‐derived measures. These methods have been showed to provide complementary information on autonomic function during sleep stages in health and disease (Vanoli et al. 1995; Tobaldini et al. 2014).

Thus, we aimed to investigate the effects of eight nights of chronic sleep restriction on ANS function during sleep in healthy subjects, using three different tools for the assessment of cardiac autonomic dynamics and complexity.

## Materials and Methods

### Experimental protocol and polysomnographic recordings

This study was a 1:1 randomized, parallel‐group study of sleep deprivation versus control sleep, stratified by sex, conducted at the Clinical Research and Trials Unit at St. Marys Hospital, part of the Center for Clinical and Translational Science (CCaTS) at Mayo Clinic (NCT01334788). The Mayo Clinic Institutional Review Board (IRB #08‐006780) approved this study. Individuals gave written informed consent. As previously described (Calvin et al. 2014), we enrolled seventeen healthy individuals, aged between 18 and 40 years, body mass index (BMI) between 18.5 and 29.9 kg/m^2^, sedentary (less than four 20‐min episodes of moderate or vigorous intensity physical activity in the prior 4 weeks), taking no medications (except for oral contraceptive pills for birth control), nonsmokers, without any overt known disease. Exclusion criteria were pregnancy, smoking, sleep disorders, anemia, and inability to follow the study protocol.

All subjects underwent a screening evaluation consisting of a physical examination, assessment of hemoglobin concentration, exercise treadmill test, urine pregnancy test, overnight polysomnography (PSG), and 1‐week actigraphy.

Within 1 month after completion of the screening examination, enrolled subjects were admitted to the Clinical Research Unit and began the 15 day/14‐night inpatient phase of the study.

The experimental protocol consisted of the following: first 3 days/3 nights as acclimation phase; experimental phase (subsequent 8 days/eight nights) and recovery phase.

As to the experimental phase, subjects were randomly assigned to the restriction group (RES; subjects were required to stay awake between 0600 h and their bedtime, which was calculated to give an in‐bed time equal to two‐thirds of their usual sleep time; *n* = 8) or Control group (CON; subjects were allowed to sleep their usual sleep time; *n* = 9).

The two groups, RES and CON, were similar in terms of demographic characteristics, such as age (mean ± SD RES vs. CON: 25 ± 4 years vs. 25 ± 5 years, *P* = 0.7), gender (CON group: six males and three females, RES group five males and three females), and BMI (22.5 ± 2.5 kg/m^2^ vs. 22.4 ± 1.2 kg/m^2^, *P* = 0.9).

Attended PSG recording included electroencephalogram, electro‐oculogram, electromyogram, electrocardiogram (ECG), and respiration via oronasal thermal airflow sensor and respiratory impedance plethysmography. ECG was sampled at 512 Hz, and the respiratory signal at 32 Hz.

PSGs recorded at the end of the acclimation phase (D3), the first night after sleep restriction (D5), at the end of the sleep restriction period (D11), and at the end of the recovery phase (D14) were selected for HRV analysis.

### Data analysis

ECG and respiratory traces (thoracic/abdominal traces) were extracted from PSG recordings to obtain consecutive segments of 250–300 beats from wakefulness (W), non‐REM sleep (N2 and N3) and REM sleep stages. Segments from the first two complete sleep cycles (sequences of NREM and REM sleep) for each subject were considered for autonomic analysis and only segments associated with stable and regular breathing, that is, the absence of central and obstructive apneas, have been used for the autonomic analysis. Segments were selected blindly to subject allocation.

HRV parameters from each segment for each sleep stage were averaged.

As to the autonomic analysis, time series of all the consecutive heart periods were derived from ECG signals. After the detection of QRS complexes, the apex of the R wave was located using a parabolic interpolation. QRS detection was checked to avoid missed beats and incorrect detection of R waves. Linear detrend was applied to all segments. Ectopic beats were identified and replaced with interpolated R‐R interval data.

#### Spectral analysis of heart rate variability

An autoregressive model was applied for spectral analysis of HRV. Ad hoc power spectral tools identified two main oscillatory components:

1. Low frequency components (LF), ranging 0.04 Hz–0.15 Hz, marker of sympathetic modulation
2. High frequency component (HF), ranging 0.15 Hz–0.4 Hz, marker of vagal modulation and synchronous with respiration.

LF and HF can be expressed both in absolute units (ms^2^) and in normalized units (nu), which represent the relative amount of each component with respect to the total power; the ratio between LF and HF, the so‐called LF/HF, is a marker of the “sympathovagal balance”.

The spectral diagram of respiratory activity comprises a unique principal component, HF RF, whose central frequency, in physiological conditions, is very close to that of the heart rate HF component (Montano et al. 2009). Using a bivariate autoregressive analysis, K^2^RR‐RESP measures the coherence between the respiratory oscillation and the cardiac cycle and is a marker of cardiopulmonary coupling; we calculate the maximum coherence at HF bands. Values range from 0 to 1, with higher values indicating a stronger coupling between heart period and respiratory oscillations.

#### Symbolic analysis

Symbolic analysis (SA) is a recently developed nonlinear tool able to detect nonreciprocal changes of sympathetic and parasympathetic modulation on heart period time series. SA is based on the transformation of time series into a sequence of symbols, the construction of patterns (i.e., words), the reduction in the number of patterns into four families, and, finally, the evaluation of their rate of occurrence.

All patterns are grouped without any loss into four families: 0V%, pattern with no variation (i.e., all the symbols are equal or in the same level), 1V % (pattern with one variation, two consecutive symbols are equal and the remaining one is different), 2LV%, patterns with two like variations (the three symbols form an ascending or descending ramp) and, finally, the 2UV%, pattern with two unlike variations (the three symbols form a peak or a valley). Physiological studies showed that 0V% is a marker of sympathetic modulation while 2LV% and 2UV% are markers of vagal modulation (Porta et al. 2007b).

#### Complexity analysis

Entropy‐derived measures provide information on the complexity of autonomic cardiac control.

Different measures have been proposed for the evaluation of autonomic complexity, such as Corrected Conditional Entropy (CCE) (Porta et al. 2007a).

CCE is based on the definition of Conditional Entropy, CE, an index that assesses the amount of information carried by the current RR sample (i.e., RR(i)) when L‐1 previous samples of RR are known (i.e., RRL‐1(i − 1) = (RR(i − 1), …, RR(i − L + 1))). This index represents the difficulty in predicting the future values of a time series when the past values are known. CCE is bounded between zero and the Shannon entropy, representing the maximum amount of information derived from the RR series. CCE decreases to 0 when the new sample is fully predictable, achieves its maximum value when the new sample is totally unpredictable, and its nadir when the knowledge of past values is helpful in reducing the uncertainty associated with future values (Porta et al. 2007a).

### Statistical analysis

SigmaStat program was used for statistical analyses. To perform the intragroup analysis, we used the ANOVA for repeated measures (ANOVA RM) with post‐hoc comparison method. Normality and homogeneity of variance residuals were tested; if the sample was normally distributed ANOVA RM and Holm‐Sidak test were performed; if this was not the case ANOVA RM on ranks and Tukey test were performed. Statistical significance was set at *P* < 0.05.

## Results

### Spectral, symbolic, and entropy analyses in the sleep restricted (RES) group

All spectral results of the RES group are reported in Table 1.

**Table 1:** Spectral analysis of HRV in RES subjects during sleep

	D3	D5	D11	D14
HR (bpm)				
W	64 ± 6^1^ ^,^ ^2^	63 ± 7^1^ ^,^ ^2^	64 ± 7^1^ ^,^ ^2^	67 ± 9^1^ ^,^ ^2^
N2	57 ± 4	58 ± 6	61 ± 6	59 ± 8
N3	58 ± 5	58 ± 5	61 ± 7	61 ± 8
REM	60 ± 5	63 ± 5^1^ ^,^ ^2^	65 ± 6^1^ ^,^ ^2^ ^,^ ^3^	66 ± 6^1^ ^,^ ^2^ ^,^ ^3^
TP (ms^2^)				
W	5039 ± 2927	5989 ± 2407	5829 ± 2250	4950 ± 2847
N2	5078 ± 2447	6725 ± 5921	4761 ± 5140	5163 ± 3197
N3	3467 ± 2137	3115 ± 1894	3149 ± 2689	3063 ± 2890
REM	5361 ± 2275	5059 ± 1983	4214 ± 2531	4558 ± 2222
LF/HF				
W	4.6 ± 5.2	2.2 ± 1.5	3.5 ± 4.1	2.3 ± 1.4
N2	2.2 ± 2.1	4.4 ± 4.7	1.7 ± 1.3	2.3 ± 2.0
N3	2.1 ± 1.9	2.3 ± 2.2	2.6 ± 3.1	1.5 ± 1.9
REM	4.6 ± 6.0	4.4 ± 4.3	7.3 ± 5.1^1^ ^,^ ^2^	4.9 ± 5.4
RESP HF (Hz)				
W	0.28 ± 0.09	0.26 ± 0.02	0.25 ± 0.02	0.27 ± 0.01
N2	0.24 ± 0.03	0.23 ± 0.02	0.27 ± 0.03	0.23 ± 0.03
N3	0.25 ± 0.03	0.24 ± 0.02	0.24 ± 0.02	0.24 ± 0.02
REM	0.26 ± 0.02	0.24 ± 0.02	0.25 ± 0.01	0.3 ± 0.03
K^2^ RR‐RESP				
W	0.76 ± 0.18^1^ ^,^ ^2^	0.67 ± 0.16^1^ ^,^ ^2^	0.75 ± 0.21^1^ ^,^ ^2^	0.81 ± 0.11^4^
N2	0.93 ± 0.04	0.88 ± 0.08	0.93 ± 0.04	0.94 ± 0.04
N3	0.94 ± 0.05	0.95 ± 0.05	0.96 ± 0.03	0.96 ± 0.03
REM	0.81 ± 0.07	0.82 ± 0.08	0.76 ± 0.12^1^ ^,^ ^2^	0.80 ± 0.12

Data are presented as mean ± SD.

*P* < 0.05: ^1^versus N2, ^2^versus N3, ^3^versus D3, ^4^versus D5.

Considering the differences between sleep stages, the RES group showed a higher heart rate (HR) during W and REM compared to N2 and N3 during D5, D11, D14. Coherence between RR time series and respiration in the HF band (K^2^ RR‐RESP), an index of cardiorespiratory coupling, was higher in N2 and N3 compared to W all days and to REM in D11. Total power, LF, HF, LF/HF, and respiratory frequency did not show any significant difference among sleep stages during all the study days.

Symbolic and entropy results in the RES group are represented in Table 2. Symbolic analysis revealed that 0V, marker of sympathetic modulation, was lower in N2 and N3 compared to W and REM on D3, it decreased in N3 compared to W and REM in D5, in N2 and N3 compared to REM at D11 and in N2 and N3 compared to W and REM at D14. On the contrary, 2LV% and 2UV% were higher in N3 compared to W and REM at D3, higher in N3 with respect to REM at D5, higher in N2 and N3 compared to REM at D11 and higher in N2 and N3 compared to REM at D14.

**Table 2:** Symbolic and complexity indices in sleep restricted subjects during sleep

	D3	D5	D11	D14
0V (%)				
W	32 ± 4^1^ ^,^ ^2^	28 ± 5^2^	24 ± 6	29 ± 5^1^ ^,^ ^2^
N2	14 ± 5	25 ± 4	18 ± 6	19 ± 5
N3	16 ± 6	15 ± 4	15 ± 5	14 ± 6
REM	30 ± 4^1^ ^,^ ^2^	40 ± 5^2^	40 ± 6^1^ ^,^ ^2^	33 ± 4^1^ ^,^ ^2^
1V (%)				
W	45 ± 3	43 ± 2	46 ± 3	46 ± 3
N2	46 ± 4	46 ± 3	48 ± 2	49 ± 3
N3	43 ± 3	45 ± 2	45 ± 3	45 ± 5
REM	44 ± 3	46 ± 3	42 ± 3	46 ± 2
2LV (%)				
W	7 ± 2^2^	10 ± 3	13 ± 4	8 ± 5
N2	15 ± 3	12 ± 4	14 ± 3	14 ± 4
N3	17 ± 2	16 ± 4	17 ± 2	18 ± 4
REM	9 ± 2^2^	8 ± 3^2^	5 ± 4^1^ ^,^ ^2^	7 ± 3^1^ ^,^ ^2^
2UV (%)				
W	14 ± 5^2^	17 ± 5	15 ± 4	14 ± 4
N2	21 ± 6	15 ± 4	18 ± 5	16 ± 7
N3	25 ± 6	22 ± 4	20 ± 5	21 ± 6
REM	15 ± 4^2^	15 ± 5^2^	11 ± 4^1^ ^,^ ^2^	12 ± 5^1^ ^,^ ^2^
CCE				
W	0.9 ± 0.1^2^	0.9 ± 0.2	0.9 ± 0.2	0.9 ± 0.1
N2	1.1 ± 0.1	1.0 ± 0.1	1.1 ± 0.1	1.1 ± 0.2
N3	1.1 ± 0.2	1.1 ± 0.2	1.1 ± 0.1	1.1 ± 0.1
REM	0.9 ± 0.2	0.9 ± 0.2	0.9 ± 0.2	0.9 ± 0.2^1^ ^,^ ^2^

CCE, Corrected conditional entropy. Data are presented as mean ± SD. ^1^versus N2, ^2^versus N3, ^3^versus REM. *P* < 0.05.

CCE was higher during N3 compared to W at D3 and higher in N2 and N3 compared to REM at D11.

### Spectral, symbolic, and entropy analyses in the Control (CON) group

All spectral results of the CON group are reported in Table 3. As to the differences among sleep stages, HR was lower in N2 and N3 compared to W, while no significant differences were observed in other spectral parameters (see Table 3).

**Table 3:** Spectral analysis of HRV in Control subjects during sleep

	D3	D5	D11	D14
HR (bpm)				
W	61.9 ± 5.9^1^ ^,^ ^2^	66.1 ± 8.0^1^ ^,^ ^2^	64.3 ± 10.0^1^ ^,^ ^2^	63.7 ± 7.0^1^ ^,^ ^2^
N2	56.8 ± 7.3	61.2 ± 8.8	57.7 ± 8.2	57.8 ± 7
N3	57.7 ± 7.1	61.8 ± 9.5	58.1 ± 7.7	57.3 ± 6.2
REM	61.3 ± 9.5^1^	62.9 ± 10.1	62.3 ± 9.2^1^ ^,^ ^2^	59.9 ± 8.2
TP (ms^2^)				
W	8250 ± 7280	5332 ± 3104	5920 ± 4479	6438 ± 3423
N2	7666 ± 8007	6159 ± 6829	7892 ± 8877	9416 ± 6151
N3	4655 ± 5612	3289 ± 3979	4117 ± 5487	4212 ± 3871
REM	8459 ± 7567	6375 ± 3673	8237 ± 8579	6973 ± 5370
LF/HF				
W	2.4 ± 1.5	2.1 ± 1.6	2.1 ± 1.5	1.9 ± 0.95
N2	2.6 ± 2.4	3.3 ± 3.8	2.5 ± 1.1	1.8 ± 1.2
N3	1.4 ± 1.2	1.6 ± 1.4	1.9 ± 1.4	1.3 ± 0.7
REM	3.6 ± 5.1	2.9 ± 2.4	2.5 ± 1.6	1.5 ± 1.2
RESP HF (Hz)				
W	0.27 ± 0.04	0.28 ± 0.02	0.24 ± 0.12	0.28 ± 0.03
N2	0.26 ± 0.01	0.26 ± 0.01	0.25 ± 0.02	0.25 ± 0.02
N3	0.26 ± 0.01	0.26 ± 0.03	0.26 ± 0.02	0.26 ± 0.02
REM	0.26 ± 0.02	0.26 ± 0.02	0.26 ± 0.03	0.26 ± 0.03
K^2^ RR‐RESP				
W	0.74 ± 0.05	0.75 ± 0.2	0.81 ± 0.07	0.77 ± 0.21
N2	0.93 ± 0.06	0.91 ± 0.1	0.93 ± 0.05	0.90 ± 0.06
N3	0.97 ± 0.02^3^	0.97 ± 0.02^3^	0.97 ± 0.03^3^	0.96 ± 0.05^3^
REM	0.80 ± 0.12	0.81 ± 0.09	0.79 ± 0.12	0.86 ± 0.10

Data are presented as mean ± SD.

*P* < 0.05: ^1^versus N2, ^2^versus N3, ^3^versus REM.

Symbolic and entropy results are summarized in Table 4. Considering symbolic and entropy measures, 0V pattern was significantly higher during W and REM compared to N2 and N3 during all study days, while on the contrary 2LV and 2UV were significantly lower during W and REM compared to N2 and N3 (see Table 4). In addition, spectral, symbolic, and complexity parameters did not show any significant difference among sleep stages across D3, D5, D11, and D14.

**Table 4:** Symbolic and complexity indices in Control subjects during sleep

	D3	D5	D11	D14
0V (%)				
W	25 ± 8^1^ ^,^ ^2^	24 ± 10^1^ ^,^ ^2^	20 ± 9^1^ ^,^ ^2^	20 ± 9^1^ ^,^ ^2^
N2	16 ± 11	22 ± 13	18 ± 8	16 ± 8
N3	9 ± 7	11 ± 8	12 ± 8	11 ± 8
REM	32 ± 18^1^ ^,^ ^2^	32 ± 17^1^ ^,^ ^2^	32 ± 14^1^ ^,^ ^2^	23 ± 14^1^ ^,^ ^2^
1V (%)				
W	43 ± 5	45 ± 6	45 ± 5	44 ± 7
N2	43 ± 4	45 ± 5	47 ± 3	48 ± 4
N3	41 ± 6	44 ± 5	44 ± 5	42 ± 6
REM	41 ± 4	42 ± 4	42 ± 4	43 ± 6
2LV (%)				
W	10 ± 4^1^ ^,^ ^2^	9 ± 4^1^ ^,^ ^2^	11 ± 3^1^ ^,^ ^2^	10 ± 5^1^ ^,^ ^2^
N2	15 ± 5	12 ± 4	12 ± 5	13 ± 4
N3	19 ± 5	18 ± 5	17 ± 6	18 ± 7
REM	9 ± 8^1^ ^,^ ^2^	8 ± 5^1^ ^,^ ^2^	9 ± 4^1^ ^,^ ^2^	12 ± 7^1^ ^,^ ^2^
2UV (%)				
W	21 ± 8	22 ± 10	24 ± 11	26 ± 11
N2	26 ± 11	21 ± 12	22 ± 8	22 ± 7
N3	30 ± 9	27 ± 11	27 ± 6	29 ± 9
REM	18 ± 12^1^ ^,^ ^2^	18 ± 10^1^ ^,^ ^2^	17 ± 9^1^ ^,^ ^2^	22 ± 8^1^ ^,^ ^2^
CCE				
W	0.98 ± 0.1^2^	0.99 ± 0.17	1.07 ± 0.14	1.06 ± 0.1
N2	1.06 ± 0.1	0.93 ± 0.1	1.03 ± 0.07	1.04 ± 0.1
N3	1.16 ± 0.1	1.10 ± 0.09	1.13 ± 0.07	1.31 ± 0.1
REM	0.97 ± 0.2^2^	0.95 ± 0.20^2^	0.96 ± 0.15^2^	1.06 ± 0.1

CCE, Corrected conditional entropy. Data are presented as mean ± SD. *P* < 0.05: ^1^versus N2, ^2^versus N3.

## Discussion

This study shows, for the first time, that after 8 days of moderate sleep restriction in healthy subjects: (1) physiological cardiac autonomic dynamics, characterized by an increased parasympathetic modulation during NREM sleep compared to W and REM, are preserved; (2) cardiorespiratory coupling increases during deep sleep compared to wake and REM, in both RES and CON groups; (3) autonomic control is less complex during REM sleep compared to NREM sleep in both RES and CON groups; (4) in the RES group, HR is significantly higher after sleep loss compared to baseline and complexity is reduced during REM with respect to baseline; (5) symbolic analysis seems to be more reliable than linear spectral analysis in tracking changes of autonomic cardiac control during different sleep stages.

Population‐based studies indicate that acute sleep deprivation is associated with increased risk of cardiovascular, cerebrovascular, and metabolic disorders (Eguchi et al. 2008; Tasali et al. 2008; Cappuccio et al. 2010). Physiologically, sleep loss affects several biological pathways (inflammation, coagulation, endothelium function, and immune response), which likely mediate the detrimental clinical consequences of sleep loss. In this setting, ANS regulation of cardiovascular function plays a key role. In fact, experimental and clinical studies showed that acute sleep deprivation importantly alters cardiac autonomic control in healthy subjects (Zhong et al. 2005; Tobaldini et al. 2013b; Sauvet et al. 2014), with a predominant sympathetic modulation, a reduction in vagal control, and a blunted response to orthostatic challenge after sleep loss. On the contrary, there is little and conflicting evidence on the effects of chronic sleep restriction in healthy subjects (Muenter et al. 2000; Dettoni et al. 2012; Calvin et al. 2014; Sauvet et al. 2015) and, to the best of our knowledge, no data on the consequences of sleep loss on cardiovascular function during sleep have been published.

Thus, in this 1:1 randomized protocol in healthy subjects, we investigated the effects of chronic sleep restriction on cardiovascular autonomic control during sleep stages using different tools for the analysis of cardiovascular autonomic control (CAC).

As to the assessment of CAC during sleep stages in the two groups, our results showed that physiological cardiac autonomic dynamics seem to be overall preserved during sleep stages. In fact, spectral and symbolic analyses showed decreased sympathetic and increased parasympathetic modulation during NREM sleep compared to W and REM in both RES and CON groups.

It is well known that sleep is a physiological process characterized by profound changes in autonomic cardiovascular control during different sleep stages (Vanoli et al. 1995; Trinder et al. 2001), with a predominant vagal modulation during NREM sleep (Somers et al. 1993; Trinder et al. 2001). We have recently shown that these autonomic dynamics may be preserved in pathological conditions, such as in patients affected by Brugada syndrome and in patients with spinal cord injuries (Tobaldini et al. 2013a,c, 2015).

Heart rate variability analysis has been widely used as a noninvasive tool for the assessment of cardiac autonomic control. Although largely accepted, some questions are still debated and no global consensus has been reached. However, our present results are in line with this prior evidence, suggesting that the physiological oscillations of sympathetic and vagal branches during the transition across different sleep stages may not be affected by chronic sleep restriction. However, we should consider that negative results we observed may be related to the duration of sleep deprivation; alternatively, it is plausible that subjects did not experience a large enough deprivation to exhibit any changes in cardiac autonomic control. Interestingly, symbolic analysis appears to be more sensitive than spectral analysis in detecting moderate fluctuations in CAC during sleep stages.

In addition, we observed that, in the RES group, HR during REM was significantly higher after experimental sleep curtailment compared to baseline, while no changes were observed during W. Previous studies on chronic sleep loss showed no effects of sleep debt on HR recorded during wakelfuness (Muenter et al. 2000; Dettoni et al. 2012); thus our data, obtained by applying a different experimental protocol, support these prior findings. However, for the first time we showed that HR was significantly higher during REM sleep after sleep loss compared to baseline, without changes of autonomic components. Hence, we speculate that sleep restriction possibly changes the autonomic offset without affecting sympatho‐vagal balance, maybe because of the high instability that characterizes this sleep stage.

As to respiration, we did not find any difference in breathing frequency as a function of sleep stage. However, we also analyzed cardiorespiratory coupling (CC) using a bivariate autoregressive analysis, which evaluates the coherence between respiratory oscillations and cardiac cycle (Dick et al. 2014). It has been shown that, in healthy subjects, CC increases from W to NREM sleep while it decreases during REM sleep (Patruno et al. 2014). In pathological conditions such as obstructive sleep apnea, CC is reduced compared to healthy subjects while effective treatment with continuous positive airway pressure (CPAP) is able to restore CC to physiological values (Chang et al. 2013; Dick et al. 2014; Patruno et al. 2014). In line with these previous reports, our data show that CC increased from W to NREM sleep and REM, in both RES and CON groups, and significantly decreased during REM in both groups. Thus, we suggest that moderate sleep loss may not influence the physiological dynamics of CC, which is a marker of the reciprocal interaction between autonomic and respiratory control systems.

Over the past years, there has been growing interest has in the application of novel techniques for the assessment of CAC. In addition to the traditional linear spectral analysis, several nonlinear methods, such as symbolic analysis and entropy‐derived measures, have been developed and validated in health and disease (Porta et al. 2007a,b), providing information complementary to that produced by linear spectral analysis.

Entropy‐derived measures yield insight into the complexity of autonomic cardiac control. Heart period is regulated by the integrative action of different mechanisms, such as central oscillators, peripheral chemoreflex and baroreflex control, molecular and local factors. A reduction in complexity implies that a certain variable is controlled by only one of such regulatory mechanisms, which may impair the ability of the system to appropriately responding to stress stimuli. During sleep, complexity of CAC changes across sleep stages. In fact, increased complexity during NREM and significant reduction during REM have been reported (Viola et al. 2011). Our data show that the dynamic of cardiac complexity is preserved after sleep restriction in this experimental protocol. In both RES and CON groups, we observed higher autonomic control complexity during NREM sleep compared to W and a significant reduction during REM compared to NREM sleep, as previously described in healthy subjects (Viola et al. 2011). However, this difference is more evident at the end of sleep deprivation period, that is, see D11. An interesting finding of our study is the observation that complexity during REM sleep is lower after sleep loss with respect to baseline. While we did not find variations in spectral and symbolic parameters after chronic sleep deprivation, complexity during REM sleep was affected. We speculate that, after chronic sleep loss, the cardiovascular vulnerability physiologically seen during REM sleep may be exacerbated during sleep deprivation, with less complex control of the cardiovascular system and, therefore, impaired ability to respond to stress stimuli.

This study has some limitations. First, we did not perform direct recordings of sympathetic nerve activity. In addition, we did not record beat‐to‐beat blood pressure, which could have added important information on cardiovascular autonomic control.

However, strengths include for the first time, evaluation of cardiac autonomic control during sleep stages after a complex experimental protocol of chronic sleep restriction. In addition, we used three different methods for the analysis of cardiac autonomic control: spectral analysis, symbolic analysis, and entropy measures, which provide complementary information on CAC.

### Perspectives

Sleep loss has become an increasingly relevant health and social problem. Sleep restriction in healthy subjects has implications for disruption of cardiovascular, inflammatory, and cognitive function. These changes at a population level will have strong relevance to the epidemiology of cardiovascular, metabolic, and neurological disease. More data are needed to understand the effects of sleep loss in individuals with established disease.

## Conflict of Interest

All authors declare no disclosure.
